# Donation or Discount: Effect of Promotion Mode on Green Consumption Behavior

**DOI:** 10.3390/ijerph18041912

**Published:** 2021-02-16

**Authors:** Jun Zou, Yifan Tang, Ping Qing, Han Li, Amar Razzaq

**Affiliations:** College of Economics and Management, Huazhong Agricultural University, Wuhan 430070, China; huanongzoujun@webmail.hzau.edu.cn (J.Z.); han.li@webmail.hzau.edu.cn (H.L.); amar.razzaq@hotmail.com (A.R.)

**Keywords:** green consumption, donation promotion, discount promotion, conceptual fluency, purchase intention

## Abstract

Environmental issues are still challenging and of global concern. To improve the environmental consumption behavior of consumers, this study investigates whether the match between the promotion mode and product type can improve the conceptual fluency of consumers, so as to increase their purchase intention for green products. The results of three experiments reveal that the interaction between promotion mode and product type has a certain impact on the conceptual fluency of consumers, which can, in turn, promote their purchase intention. This research theoretically contributes to the research on green consumption by introducing promotion mode and revealing the mediation effect of conceptual fluency, it also provides some practical implications for alleviating environmental problems.

## 1. Introduction

Solving global environmental problems is still full of challenges, which are closely associated with consumer ignorance or disapproval of green products. Green products refer to products with certain environmental attributes, for example, products made from recycled or biodegradable materials [1,2]. Although the sales of green products have reached $845 billion, more than half of consumers still choose not to purchase green products because their perceived quality is relatively low [3]. Therefore, it is crucial to continuously and substantially promote the green buying behavior of consumers.

Extensive research has focused on the factors that influence consumers’ purchase intentions of green products. Most of them explained green consumption behavior from the perspective of consumers themselves [4,5,6,7,8]. Besides, some researchers have found that consumers’ green product purchase intentions can be affected by environmental factors, such as product eco-labels [9] and carbon label placement [10]. However, promotion mode, an extremely important factor in product sales, has been largely ignored in previous studies. A recent study compared the effect of discount promotion and bonus pack promotion on green products [11]. However, no study has tested the effectiveness of donation promotion on consumer buying behavior toward green products. Evidence shows that donation promotion can activate consumer cause-related social identity [12]. Since green product is the symbol of altruism due to its environmental attributes, therefore, we predict that donation promotion mode can be a more effective strategy for green product marketing. Drawing from fluency theory [13,14], we speculate that donation promotion may match green products better. In this context, we aim to investigate whether consumer purchase behavior differs across different promotion modes of green products.

Across three experiments, the present study investigated the interactive effect of promotion mode and product type on consumer purchase intention and further explored which promotion mode is more suitable for promoting green products. We demonstrated that compared with discount promotion, donation promotion can have a more positive influence on consumer purchase intentions by increasing conceptual fluency during the purchase. 

This study may have several theoretical and managerial contributions. First, the current research advances our understanding of how to increase consumer purchase intention of green products. Although past research on green products has found some drivers of green product purchases [4,5,9,10,15,16,17], there is limited research on how to promote green products from the perspective of promotion mode. In addition, recent research has only explored the match between promotion mode and consumers’ self-construal tendency [12]. The current research finds the relationship between promotion mode and product type and shows that when the donation promotion is used for promoting green products, the expression of altruism can be more fluent. In this sense, the current research contributes to past research by demonstrating a positive effect of using donation promotion on promoting green products.

Secondly, the current research contributes to an extensive understanding of fluency theory. We reveal the mechanism underlying the match between promotion mode and product type and demonstrate that only when the green product is promoted with a donation promotion will the conceptual fluency during purchase be higher, which, in turn, leads to higher purchase intention. Managerially, our findings may facilitate the choice of the appropriate promotion mode for promoting the consumption of green products, for example, when a seller wants to promote a pair of glasses made from recycled material, they can adopt a donation promotion (donating one percent of the price to a nonprofit organization), instead of offering a direct discount to consumers. Besides, the government can also raise altruism appeal when advocating some green behaviors, which may contribute to the alleviation of environmental problems.

The remainder of the article is organized as follows. In the next section, we review the previous research on green consumption to list the existing drivers promoting green consumption. Then, we introduce promotion mode and develop the hypotheses of current research. Subsequently, we describe the three experiments in which we test our hypotheses empirically. Finally, we discuss the theoretical and managerial implications of the findings.

## 2. Theoretical Background and Hypotheses

### 2.1. Green Consumption

With the growing popularity of green consumption worldwide, the academic community has also paid increasing attention to environment-friendly consumption behaviors. Most of the previous research has explained green consumption behavior from the perspective of consumers themselves. Ramayah and Lee (2010) [4] used the Theory of Reasoned Action (TRA) to reveal that individual values and attitudes can influence green product purchase intention. Another research that was based on the Theory of Planned Behavior (TPB) suggested that green purchase attitudes, subjective norms, and perceived behavior control can promote green purchase intention [5]. Further, Kamonthip et al. (2016) [6] extended the framework of TPB and found that environmental concern can have a positive effect on attitude, perceived behavioral control, and purchase intention for green products. Based on this research, Zhang et al. (2019) [8] found that product type had a moderation effect on functional products (vs. hedonic products) and the effect of environmental concern on green product purchase intention was stronger. Arli and Tan (2018) [7] proposed that consumer attitude, perceived behavioral control, pro-environmental self-identity, and ethical obligation may positively influence sustainable consumption through readiness to be green.

There is also a significant amount of literature that focused on consumers’ beliefs and skepticism of green products. In the 1990s, many companies tried to capitalize on the trend of the consumption of green products and rather misled consumers by selling unchanged products as eco-friendly products. However, any short-term gains they received were offset by a long-term consumer distrust [18]. Consequently, widespread skepticism among consumers keeps them from purchasing green products even when the demand is strong. Further, those who purchase green products are not sure whether they are truly helping the environment. Today, greenwashing has become a common term for the companies that mislead consumers about the environmental practices of a company [19]. Therefore, marketers should not blindly use green product promotion strategies and those who do so must be willing to subject their business to further scrutiny [20].

Despite consumer cynicism about green products, it is a promising industry and keeps attracting the attention of researchers. In recent years, increasing research on consumer behavior has been focused on how environmental factors, instead of consumer perception or characteristics, affect green consumption intent or behavior. When consumers perceive eco-labels as credible, they would prefer products with an eco-label [9]. Similarly, consumer green advertising receptivity also positively affects their intention [15]. Zhou et al. (2019) [10] found that when a carbon label was put on the right side, the intention for green products was higher. For millennials, online product reviews and self-image congruence had an interplay effect on green consumption behavior [17]. Additionally, Sun (2020) [15] suggested that social media marketing positively affected subjective norms and product knowledge, which, in turn, increased purchase intention for green products. However, green advertising skepticism on social media may reduce this preference [16].

Although previous research has investigated how green packaging influences green consumption from the perspective of the sellers, the depth and width are far from enough for the urgent need of improving the environment. However, as the most effective way to increase sales, the promotion has received little research attention so far. Tripathi and Pandey (2019) [11] investigated the influence of promotion modes on the purchase intention of green products. However, they divided green products into hedonic green products and utilitarian green products. Compared to nongreen products, the green product must have some environmental attributes [1,2], which indicates the pursuit of altruism. Hence, only when the promotion mode can match the green product in altruism will the promotion be the most effective, which may lead to increased intentions for green consumption.

In addition, previous research compared the effects of discount promotion and bonus pack promotion on green products [11]. Nevertheless, research in the promotion field usually uses donation promotion versus discount promotion [12,21]. Thus, the present study aims to investigate how promotion mode (discount promotion vs. donation promotion) influences the green product purchase intention of consumers.

### 2.2. Donation Promotion vs. Discount Promotion

Since sales promotions account for a large proportion of the marketing budget of corporates (more than 50% in some cases) [22] and discount promotion is the most classic promotion mode, much research has been carried out to explore the effectiveness of the discount promotion. Although discount promotion can effectively improve some important market indicators such as sales and customer flows [23], it has some negative effects on price sensitivity and brand equity [24]. These potential negative effects also prompt managers to consider alternative strategies for discount promotion, such as donation promotion, which means that the purchase of consumers will be coupled with a certain donation from the store/company to charity. Similar to traditional promotions, donation promotion can increase sales [25] and at the same time evade the negative effects of discount promotion [26,27].

Spending on donation promotion is rising year by year, from about $100 million in 1990 to more than $1.5 billion in 2009 [28]. The increasing popularity of donation promotion in the market is supported by its unusual effectiveness: 80% of consumers responded that when prices and quality are the same between products or brands, they are more willing to choose those that support philanthropy [21]. However, when there are differences in prices and quality between the products, only 19% of consumers said that they are willing to buy goods at higher prices to support philanthropy [21], suggesting that discount promotion is still preferred by most consumers.

### 2.3. Promotion Modes, Product Types, and Conceptual Fluency

As previously stated, the major difference between the two types of promotion is that the traditional discount promotion can bring more favorable results to consumers, while the donation promotion can bring more benefits to society (Arora and Henderson, 2007). In addition, Winterich and Barone (2011) [12] demonstrated that dependent self-construal consumers are more likely to choose donation promotion than independent self-construal consumers, indicating that consumers who choose donation place more value on altruism and pay more attention to the occurrence of altruistic behavior, while those choosing discount promotions are more inclined to prefer self-interest or nonaltruistic behavior.

Moreover, different product types not only have their intrinsic attributes but also bear different social or symbolic values. Consumers may express their concern or goodwill to the environment and society through the purchase of green products. In this way, green products are endowed with a certain social value, namely, altruism. Compared with that of nongreen products, the purchase of green products can satisfy the altruistic need of consumers.

The reasons for donation promotion outperforming discount promotion in the sales of green products remain largely unknown. Previous studies of conceptual fluency may provide some explanations for this effect [13,29,30]. A focal object will be evaluated more favorably if it is easier for individuals to interpret its meaning—namely, conceptual fluency [31]—which may lead to a better promotion effect. Conceptual fluency can be enhanced by activating the target stimulus in memory through the repeated presentation of the same stimulus, a semantically equivalent or related item, or a sentence context that is predictive of the target item [13].

Therefore, if the seller takes donation promotion rather than discount promotion for green products, the pro-environmental altruistic behavior represented by the consumers’ green consumption behavior can be well-matched with the altruistic psychology inspired by the donation promotion, which will enhance the consumers’ conceptual fluency during the purchase of green products. However, the purchase of an ordinary commodity does not show the motivation of altruistic behavior. Hence, whether it be donation promotion or discount promotion, altruistic behavior cannot be matched with altruistic psychology. Based on this discussion, we propose the following hypothesis:

**Hypothesis** **1 (H1).** *The interaction between promotion modes and product types has a certain effect on conceptual fluency. Specifically, for the purchase of green products, the discount promotion will contribute to a higher conceptual fluency than donation promotion.*

### 2.4. Mediating Role of Conceptual Fluency

Several studies have verified that consumers’ product evaluations can be affected by their conceptual fluency [32,33]. Whether information cues can be processed more smoothly depends on their conceptual consistency with the presented scenario (such as the activated target or concept) [34]. This matching effect has been found in brand evaluation [14], product selection [35], and consumer evaluation [36].

In addition, previous studies have found that in a consumer-related situation, the subjects can produce a more positive attitude towards the product when the mind is more likely to produce a certain goal, either because its precursors are presented or it is activated by the relevant concepts [13].

Therefore, when consumers are carrying out green consumption, donation promotion can improve the conceptual fluency in the purchase process, which, in turn, enhances the consumers’ willingness to buy green products. Thus, we propose the following hypotheses.

**Hypothesis** **2 (H2).** *The promotion mode and product type have an interactive effect on consumers’ purchase intention. Specifically, in terms of green products, donation promotion will lead to a higher purchase intention of consumers than discount promotion.*

**Hypothesis** **3 (H3).** *Conceptual fluency mediates the interactive effect.*

**Figure 1:**
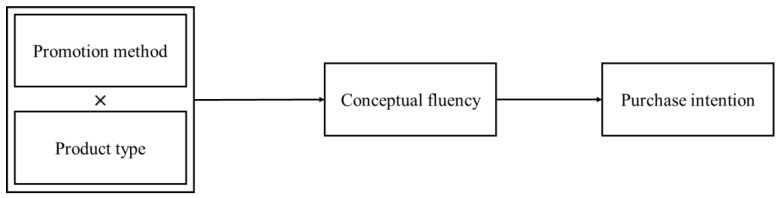
Conceptual model.

Figure 1 presents a conceptual framework of current research and the general theoretical relationships of variables.

## 3. Methods

A total of three experiments, including a field experiment (experiment 1), a laboratory experiment (experiment 2), and an online experiment (experiment 3), were carried out in this study. Experiment 1 explores the effect of the interaction between promotion modes and product types on the purchase intention of consumers. Experiment 2 further investigates the mediation effect of conceptual fluency in the model. Experiment 3 is an online experiment to test the underlying mechanism through manipulating the level of conceptual fluency, with the aim to enhance the robustness of the research.

### 3.1. Experiment 1

Experiment 1 explores the impact of promotion methods on consumers’ purchase intentions under different product types. We predict that donation promotion can better match with green products, that is, a donation promotion can contribute to higher purchase intention of green products than a discount promotion (H2).

#### 3.1.1. Design and Procedure

Experiment 1 employed a 2 (donation promotion vs. discount promotion) × 2 (green product vs. common product) between-subjects design.

The experiment was conducted in the streets and at the entrance of supermarkets of Wuhan, and the participants were the citizens of Wuhan. One hundred and eighty-five subjects completed the full procedure (M_age_ = 31.34; female, 49.73%), they were randomly divided into four scenarios. In this experiment, biodegradable disposable paper cups were selected as the green product, while common disposable paper cups were used as the ordinary product. First, the subjects were asked to imagine that they were going to entertain friends at home that night, while the disposable paper cups had been used up; thus, they were planning to buy some (biodegradable) disposable paper cups. In the scenario of donation promotion, the participants were told that “*for the purchase of every set of (biodegradable) disposable paper cups, the supermarket will donate 0.1 yuan to Hope Primary Schools*”; in the scenario of discount promotion, they were told that “*for the purchase of every set of (biodegradable) paper cups, the price will be reduced by 0.1 yuan*” [12]. Each subject was presented with a disposable paper cup, and the research assistant would tell them whether the paper cup was an environment-friendly product and which promotion mode was used. After the information was presented and understood, the participants completed the manipulation check (such as “*the disposable paper cup you will purchase belongs to eco-friendly products*”, with 1 = completely disagree and 7 = fully agree), measurement of purchase intention (such as “*I am likely to purchase this disposable paper cup*”, with 1 = strongly disagree and 7 = strongly agree, α = 0.95), and measurement of demographic variables. At the end of the task, the research assistant thanked the subjects for their participation.

#### 3.1.2. Data and Results

***Manipulation check.*** The participants agreed that biodegradable disposable paper cups were more environment-friendly than ordinary disposable paper cups (M_degradable_ = 5.47, SD = 1.43; M_ordinary_ = 4.15, SD = 1.78; t = 30.65, *p* < 0.001).

**Figure 2:**
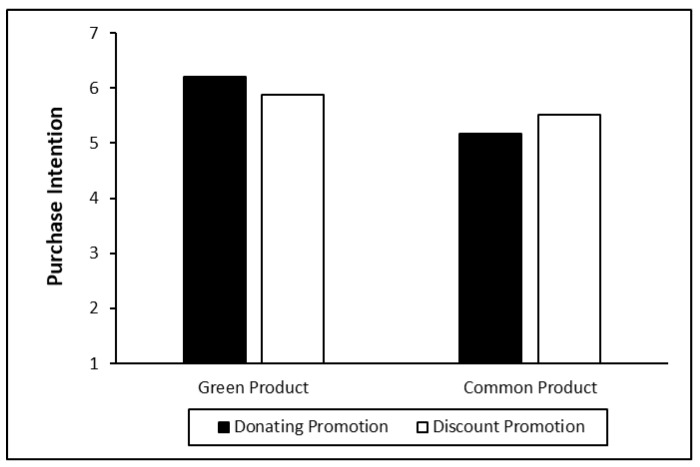
Consumers’ purchase intentions on green products vs. common products under different promotion modes.

***Purchase intention.*** In order to test the effect of the interaction between promotion mode and product type on purchase intention, we conducted an ANOVA on purchase intention with promotion mode and product type as the independent variables. As a result, a significant interaction effect (F = 6.10, *p* = 0.014) was found. In the scenario of green products, compared to discount promotion, donation promotion can lead to marginally higher purchase intention (M_donation_ = 6.20, SD = 0.64; M_discount_ = 5.88, SD = 0.76, F = 2.78, *p* = 0.097). However, for ordinary products, discount promotion can lead to marginally higher purchase intentions (M_donation_ = 5.17, SD = 1.20; M_discount_ = 5.52, SD = 0.94, F = 3.32, *p* = 0.070; Figure 2).

#### 3.1.3. Discussion

The results in experiment 1 reveal that the product type and promotion mode have an interactive effect on purchase intention. Specifically, when consumers plan to buy a green product, donation promotion can contribute to a higher purchase intention than discount promotion; nevertheless, when they consider buying a common (nongreen) product, the discount promotion has a better effect than donation promotion, which supports H2.

However, experiment 1 only investigates and validates the interactive effect of the promotion mode and product type on purchase intention, while the mechanism underlying their interactive effect on purchase intention remains to be tested. Hence, in experiment 2, we further explore and verify how conceptual fluency mediates their interactive effect.

### 3.2. Experiment 2

Experiment 2 was conducted with two purposes. The first is to verify H2 and ensure the robustness of the research; the second is to investigate the mediation effect of conceptual fluency on the interactive effect of promotion mode and product type (H1).

#### 3.2.1. Design and Procedure

Experiment 2 is a laboratory experiment, which adopts a 2 (donation promotion vs. discount promotion) × 2 (green product vs. common product) between-subjects design.

We recruited college students and residents of Wuhan, and a total of 174 subjects participated in the experiment (M _age_ = 23.10; female, 60.92%). The subjects involved in the experiment were randomly divided into four scenarios. In this experiment, phosphorus-free laundry liquid was selected as the green product, while ordinary laundry liquid was used as the common (nongreen) product. First, the subjects were asked to imagine that when washing clothes with washing machines, they discovered that the laundry liquid was running out, so they opened the Taobao App (the largest online shopping platform in China), and were ready to buy some laundry liquid. The donation promotion was manipulated as “*for the purchase of every bottle of (phosphorus-free) laundry liquid, the seller will donate 0.1 yuan to the Project Hope*”, while the discount promotion was manipulated as “*for the purchase of every bottle of (phosphorus-free) laundry liquid, the seller promises to automatically deduct 0.1 yuan when paying*” (Winterich and Barone, 2011). Each of the subjects would be presented with one type of laundry liquid and a promotion method. Then, the participants completed the manipulation check (such as “*The laundry liquid you will purchase is an environment-friendly product*”, with 1 = completely disagree and 7 = fully agree), measurement of conceptual fluency (such as “*How do you think the seller’s promotion is suitable for the product*”, with 1 = completely disagree and 7 = fully agree, ), measurement of purchase intention (such as “*I am likely to purchase this laundry liquid*”, with 1 = strongly disagree and 7 = strongly agree; α = 0.93), and the measurement of demographic variables. At the end of the test, the lab assistant thanked the subjects for their participation.

#### 3.2.2. Data and Results

***Manipulation check.*** The participants felt that phosphorus-free laundry liquid is more environment-friendly than ordinary laundry liquid (M_phosphorus-free_ = 5.43, SD = 0.97; M_ordinary_ = 4.59, SD = 1.33; t = 22.48, *p* < 0.001), indicating that manipulation of the donation promotion mode was successful.

***Purchase intention***. Similar to experiment 1, we conducted an ANOVA with purchase intention as the dependent variable and the product type and promotion mode as the independent variables. The results reveal that the interaction between product type and promotion mode has a significant effect on purchase intension (F = 5.82, *p* = 0.017). To further test the interactive effect, we conducted a simple effect analysis and found that in the scenario of green products, donation promotion can lead to higher purchase intention than discount promotion (M_donation_ = 5.70, SD = 0.86; M_discount_ = 4.76, SD = 1.11, F = 15.51, *p* < 0.001), while in the scenario of common (nongreen) products, there is no significant difference between donation promotion and discount promotion (M_donation_ = 4.89, SD = 1.36; M_discount_ = 4.76, SD = 1.09, F = 0.28, *p* = 0.601), which supports H2.

**Figure 3:**
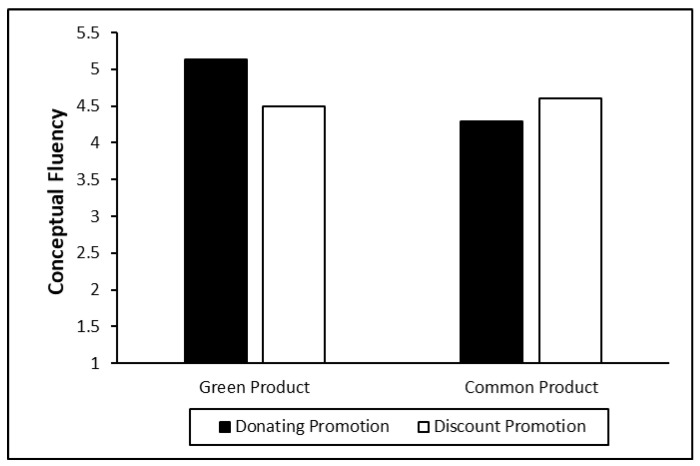
Conceptual fluency for green products vs. common products under different promotion modes.

***Conceptual fluency***. For the analysis of conceptual fluency, we conducted another ANOVA with conceptual fluency as the dependent variable and product type and promotion mode as the independent variables. There was an interaction effect with conceptual fluency (F = 7.31, *p* = 0.008). In the scenario of green products, donation promotion leads to a higher conceptual fluency than discount promotion (M_donation_ = 5.13, SD = 0.91; M_discount_ = 4.50, SD = 1.09, F = 6.48, *p* = 0.012); in the scenario of common (nongreen) products, there is no significant difference between the two promotion modes (M _donation_ = 4.29, SD = 1.23; M_discount_ = 4.61, SD = 1.31, F = 1.63, *p* = 0.20), which supports H1 (See Figure 3).

***Mediation effect of conceptual fluency.*** In order to verify the mediating role of conceptual fluency, we inserted a new variable into the analysis, namely, product type × promotion mode. Then, a bootstrap mediation analysis [37] was conducted using 1000 resamples, with the product type × promotion mode interaction as the independent variable, conceptual fluency as the mediator, and purchase intention as the dependent variable. The results showed that the match between green products and donation promotion could lead to a higher conceptual fluency, which, in turn, promotes the purchase intention, namely, the mediation effect of conceptual fluency (b = 0.10, CI: 0.17, 0.58). These results support H3.

#### 3.2.3. Discussion

This experiment first testifies the main effect to ensure the robustness of results and then demonstrates the interactive effect of product type and promotion mode on purchase intention (H2). We first explored the interactive effect on conceptual fluency (H1) and the mediation effect of conceptual fluency (H3). Next, to increase the robustness of our conclusion, we manipulate the mediator conceptual fluency to indirectly explore the influence of promotion mode on purchase intention and the underlying mechanism.

### 3.3. Experiment 3

In this experiment, we manipulated the conceptual fluency to a high level in different scenarios and investigated whether the effect of the promotion mode on the purchase intention is affected by conceptual fluency, to indirectly verify the mediating role of conceptual fluency. According to our hypothesis, it can be concluded that the difference in promotion mode can lead to the variation in conceptual fluency only when the product type is a green product. Therefore, in this experiment, we focused on the green product to investigate the influence of promotion mode on purchase intention under a higher level of conceptual fluency.

#### 3.3.1. Design and Procedure

Experiment 3 is based on an online survey, which employs a 2 (donation promotion vs. discount promotion) × 1 (green product) between-subjects design.

This experiment recruited 207 participants from Wenjuanxing (M _age_ = 30.17; female, 59.9%). These subjects were randomly divided into two scenarios. Similar to experiment 2, we also selected phosphorus-free laundry liquid as the green product and the scenario is the same as experiment 2 except that phosphorus-free laundry liquid (green product) is the only tested product. The manipulation of donation promotion is “*for the purchase of every bottle of phosphorus-free laundry liquid, the seller promises to donate 0.1 yuan to the Project Hope*”, while that of the discount promotion is “*for the purchase of every bottle of phosphorus-free laundry liquid, the seller promises to automatically deduct 0.1 yuan when paying*” (Winterich and Barone, 2011). Different from experiment 2, this experiment not only manipulates the promotion mode and limits the product type to green products, but also activates the participants’ conceptual fluency to a relatively high level. Thus, we told the participants that the promotion adopted by the seller is the most suitable for this phosphorus-free laundry liquid. After manipulation, the participants completed the manipulation check of product type and conceptual fluency similar to the previous experiment and completed the measurement of purchase intention. Upon completion of the test, we thanked the subjects for their participation.

#### 3.3.2. Data and Results

***Manipulation check.*** This experiment manipulates two variables (promotion mode and conceptual fluency) and focuses on one variable (product type). ANOVA reveals that there is no significant difference in perceived environmental friendliness for the green product between the two promotion modes (M_donation_ = 5.43, SD = 1.20; M_discount_ = 5.63, SD = 1.04; t = 0.26, *p* = 0.609). As for conceptual fluency, there is also no significant difference between the two promotion modes (M_donation_ = 4.79, SD = 1.02; M_discount_ = 4.47, SD = 1.13; t = 0.22, *p* = 0.638). Therefore, the manipulation was successful.

***Hypothesis test.*** As we artificially manipulated the conceptual fluency to a relatively high level—that is, conceptual fluency does not vary between the two promotion modes—and the high-level conceptual fluency leads to high purchase intention, the difference in promotion mode cannot lead to the variation in purchase intention through the mediation effect of conceptual fluency. The ANOVA results confirmed that there is no significant difference in purchase intention between the two promotion modes (M_donation_ = 5.40, SD = 1.10; M_discount_ = 5.24, SD = 1.01; t = 1.02, *p* = 0.313), which indirectly validates the mediating role of conceptual fluency in the relationship between promotion modes and the purchase intention of green products.

#### 3.3.3. Discussion

This experiment manipulates the mediator to indirectly verify the mediation effect of conceptual fluency in the whole model. Thus, it can be concluded that the interaction between product type and promotion mode can have a significant effect on purchase intention under the mediation of conceptual fluency.

Overall, our three hypotheses were repeatedly validated in the three different experiments.

## 4. General Discussion

### 4.1. Theoretical Contribution

Although many studies have reported that green consumption behavior is influenced by various factors, most of the research is focused on the application of the Theory of Planned Behavior [6,7,8,15,38,39] and the Theory of Reasoned Action [4]. In addition, other researchers investigated the influence of individual values [4] and environmental concern [6] on consumer green consumption. From the perspective of advertising, Sun and Wang (2020) [15] found that social media can positively affect intention, while green advertising skepticism may reduce it [16]. However, most of the previous research only explained why consumers prefer or refuse green products from the point of consumers themselves, instead of investigating what method or advertising enterprises can use to promote green products.

Thus, this study may have several theoretical and managerial contributions. First, the current research advances our understanding of how to increase consumer purchase intention of green products. Although past studies on green products have compared the effect of discount promotion and bonus pack promotion on green product purchasing [11], there is still only limited research investigating how to promote green products from the perspective of promotion mode. In addition, recent research explored the match between promotion mode and consumer self-construal tendency [12] or green product type [11]. The current research finds the relation between promotion mode and product type and shows that when the donation promotion is used for promoting green products, the expression of altruism can be more fluent, which, in turn, will increase the purchase intention of green products. In this sense, the current research contributes to past research by demonstrating a positive effect of using donation promotion for promoting green products.

Further, the present research also reveals the mechanism of the positive effect of donation promotion on green product purchase intention. When the promotion method matches the product type, that is, when donation promotion is used to promote the green product, consumer conceptual fluency during purchasing will be strengthened, which, in turn, may increase purchasing intention. In fact, our research empirically demonstrates the influence of the interplay of promotion method and product type on purchase intention and that conceptual fluency can mediate the relationship between the interaction and purchase intention.

### 4.2. Practical Implications

Managerially, our findings may facilitate the choice of appropriate promotion mode for promoting the consumption of green products, for example, when a seller wants to promote a pair of glasses made from recycled material, they can adopt a donation promotion (donating one percent of the price to a nonprofit organization), instead of offering a direct discount to consumers. Besides, the government can also raise altruism appeal when advocating some green behaviors, which may have some contributions to the alleviation of environmental problems.

In addition, whether it be the proposal of research questions or the selection of independent variables, our research is based on the real phenomenon that product type moderates the influence of promotion mode on purchase intention. More importantly, both variables are extremely manipulable, making it easy for enterprises to utilize our findings when designing their promotion strategies and selecting promotion products.

However, it should be noted that enhancing sales of green products using more effective marketing strategies without a balanced approach may be contradictory to the idea of ‘green’ consumption itself. It must be coupled with a proportional decrease in the consumption and production of nongreen products to have a positive impact on the environment. Otherwise, the promotion of green products may end up doing more harm than their intended benefits. Furthermore, standards and labels of green products should be clearly specified to ensure that product is really *green* and environment friendly, and consumers’ awareness about these issues should be enhanced. In the context of this research, this would also serve for better conceptual fluency.

### 4.3. Limitations and Future Research

We have tried our best to make our research as rigorous as possible in the theoretical aspects, but there are still several limitations, which may indicate some directions for future research. Firstly, this research is only specifically focused on the interactive effect of product type and promotion mode on conceptual fluency, particularly in the scenario of green products. Thus, it remains to be determined whether there are some mediators in the scenario of nongreen products that can decide which promotion mode is better.

Secondly, although we conducted three experiments with standard procedures, our research still lacks a field study to apply and testify to our findings under real circumstances. Therefore, a future field study may be conducted to further increase the robustness and validity of this research.

Thirdly, our three experiments were all conducted in China, thus, participants are Chinese. It is inevitable to ignore the potential influence of culture on consumer green consumption. Perhaps, for consumers with different cultures or ideologies, the conclusion of current research may have some changes. Hence, future research can introduce culture, value, and ideology as moderators or control variables to investigate the culture boundary of our conclusion.

Fourthly, although we manipulated the levels of conceptual fluency, we did not test our hypotheses while considering consumers’ heterogeneity in terms of their pro-environmental beliefs. It may be possible that the effects of promotion modes also vary across types of consumers, i.e., pro-environmental vs. other consumers. So, it also remains a potential area for further research.

Finally, although the current research manipulated the promotion mode according to past research [12,40], the adaptation of manipulation calls for further research. In addition, the relevance between the organization that receives the donation and the industry of product may affect our conclusion, which is also worthy of investigation. In this research, we considered consumers’ conceptual fluency regarding altruistic attributes embodied in green products and donations to charity (Hope Project). However, conceptual fluency may also be enhanced by other attributes of green products, which might be linked to different kinds of donation promotions. So, we leave the task of studying these aspects task to future researchers.

## Data Availability

The data are not publicly available to protect the confidentiality of the participants.
